# Heterogeneous distribution of trastuzumab in HER2-positive xenografts and metastases: role of the tumor microenvironment

**DOI:** 10.1007/s10585-018-9929-3

**Published:** 2018-09-08

**Authors:** Jennifer Hazel Elizabeth Baker, Alastair Hugh Kyle, Stefan Alexander Reinsberg, Firas Moosvi, Haley Margaret Patrick, Jordan Cran, Katayoun Saatchi, Urs Häfeli, Andrew Ivor Minchinton

**Affiliations:** 1 Integrative Oncology - Radiation Biology Unit, BC Cancer Research Centre, 675 West 10th Ave, Vancouver, BC V5Z 1L3 Canada; 2 Department of Physics & Astronomy, University of British Columbia, Vancouver, Canada; 3 Department of Pharmaceutical Sciences, University of British Columbia, Vancouver, Canada

**Keywords:** Herceptin, Monoclonal antibody therapeutic, HER2/neu, Bevacizumab, DCE-MRI, Tumor microenvironment, Tumor vessel permeability, Drug distribution, HER2-positive metastases, Brain metastases

## Abstract

Most HER2-positive metastatic breast cancer patients continue to relapse. Incomplete access to all target HER2-positive cells in metastases and tumor tissues is a potential mechanism of resistance to trastuzumab. The location of locally bound trastuzumab was evaluated in HER2-positive tissues in vivo and as in vivo xenografts or metastases models in mice. Microenvironmental elements of tumors were related to bound trastuzumab using immunohistochemical staining and include tight junctions, vasculature, vascular maturity, vessel patency, hypoxia and HER2 to look for correlations. Trastuzumab was evaluated alone and in combination with bevacizumab. Dynamic contrast-enhanced magnetic resonance imaging parameters of overall vascular function, perfusion and apparent permeability were compared with matched histological images of trastuzumab distribution and vascular patency. Trastuzumab distribution is highly heterogeneous in all models examined, including avascular micrometastases of the brain and lung. Trastuzumab distributes well through the extravascular compartment even in conditions of high HER2 expression and poor convective flow in vivo. Microregional patterns of trastuzumab distribution in vivo do not consistently correlate with vascular density, patency, function or maturity; areas of poor trastuzumab access are not necessarily those with poor vascular supply. The number of vessels with perivascular trastuzumab increases with time and higher doses and dramatically decreases when pre-treated with bevacizumab. Areas of HER2-positive tissue without bound trastuzumab persist in all conditions. These data directly demonstrate tissue- and vessel-level barriers to trastuzumab distribution in vivo that can effectively limit access of the drug to target cells in brain metastases and elsewhere.

**Electronic supplementary material:**

The online version of this article (10.1007/s10585-018-9929-3) contains supplementary material, which is available to authorized users.

## Background

Treatment options for patients with the aggressive HER2-positive form of breast cancer continue to improve, though metastatic breast cancer remains a largely incurable disease. Brain metastases are of particular importance in HER2-positive breast cancer patients, as with improved treatments and prolonged survival the incidence of brain metastases as the first evidence of relapse has increased [1, 2]. This effect has been attributed to the phenomenon of the blood–brain barrier (BBB) creating a sanctuary site by preventing drug access [3, 4]. Antibody-based therapeutics such as trastuzumab are proposed to have difficulty crossing the BBB and therefore brain metastases evade drug activity [1, 5, 6]. In addition to the BBB, specific characteristics of the tumor microenvironment, beginning with a relative paucity of functional vessels, can thwart the access of drugs to their targets such that a population of under-exposed cells may survive and repopulate the tumor [7]. We have demonstrated that many small molecule cytotoxics have limited tumor tissue penetration in both in vivo and in vivo model systems due to difficulties with drug supply, flux through tissue, consumption or sequestration by cancer cells close to blood vessels [8–12].

Macromolecular compounds such as monoclonal antibodies (MAbs) face particular extravascular distribution difficulties due to their high molecular weights and target-binding affinity [13, 14]. The relatively slow distribution of MAbs has been attributed to the binding site barrier hypothesis (BSBH), where in the case of high affinity binding of the MAb to antigen, MAb distribution is limited by its binding in the presence of ample antigen [15]. Data from our lab in the vector overexpressed MDA-435-LCC6^HER2^ model showed that despite the ability of trastuzumab to distribute far from vessels in tumors with relatively even HER2 distribution, there persisted HER2-positive tissue with poor access to trastuzumab after peak plasma exposures [16]. Heterogeneity is very striking at the vessel level, as trastuzumab is able to extravasate from only a subpopulation of perfused vessels. Other groups have also demonstrated a limitation on the accumulation of trastuzumab in tumors, focusing on net tumor accumulation [13, 14, 17].

The ability of a drug to access and affect all its target cells is intuitively crucial for treatment success. The relatively slow distribution of MAbs through the interstitium in solid tumors is recognized, but the long half-life of MAbs and prolonged plasma exposure is expected to ensure adequate time to access all the tissues in most treatment scenarios. However, we have found that in addition to distributing slowly, MAbs experience additional barriers, leaving some areas of tissue with inadequate drug exposure. The aim for this study was to use HER2-positive tumor and metastases models of cancer to determine potential limits to trastuzumab access that may represent a mechanism of resistance to targeted therapies. The microenvironments of HER2-positive tumors and metastases are examined in detail using immunohistochemistry maps, 3D tissue models and dynamic imaging of tumor vasculature.

## Methods

### Reagents

Trastuzumab and bevacizumab (Roche, Genentech) were provided by the British Columbia Cancer Agency pharmacy; dilutions to 1–2 mg/ml were prepared in sterile 0.9% NaCl before intra-peritoneal (i.p.) injection. Human isotype control IgG1 (Sigma) was administered from similar concentrations. Trastuzumab and IgG antibodies for use in combination with bevacizumab were tagged with fluorescent labels according to Alexa Fluor 546 Protein Labeling Kit (ThermoFisher) instructions. Hypoxia marker pimonidazole (Hypoxyprobe) was administered at 60 mg/kg as an i.p. injection 2 h prior to tissue harvest. Fluorescent dye DiOC_7_(3) (Molecular Probes), 0.6 mg/ml dissolved in 75% (v/v) dimethyl sulfoxide/25% sterile H_2_O, was administered intravenously as a marker of vessel perfusion 5 min prior to tissue harvest [18].

### Cells and 3D tissue discs

HER2-positive breast BT474 and MDA-MB-361 and ovarian SKOV3 carcinoma cells were obtained from the American Type Culture Collection and used from early passage; JIMT-1 cells were kindly provided by Dr. Wieslawa Dragowska (BC Cancer Research Center, Vancouver). MDA-MB-231-BR-HER2 cells transfected using pCMV4.ErbB2 full-length human cDNA and pSVzeo were provided by the laboratory of Dr. Patricia Steeg at the National Cancer Institute; stable HER2-positive clones were selected using 500 µg/ml zeocin (Invitrogen). All cells were maintained as monolayers using minimum essential media, MEM/EBSS (HyClone) supplemented with 10% fetal bovine growth serum (HyClone). Multi-layered 3D tissue discs were grown on polytetrafluoroethylene (PTFE) membrane inserts as previously described [11], in media reservoirs with vigorous stirring to maintain constant distribution of O_2_, nutrients and waste materials. MAbs were added to reservoirs at 50 µg/ml, discs were removed for freezing as indicated.

### Mice and tumors

Female NOD-SCID mice weighing 20–28 g between 8 and 16 weeks of age were bred and maintained in our institutional pathogen free animal facility. Mice were implanted with 60 day 17-β-estradiol pellets (Innovative Research of America) subcutaneously 3 days before implantation of BT474 or MDA-MB-361 tumors. Tumors were implanted as single cell suspensions (2–10 × 10^6^ cells) into the subcutaneous sacral region or into the inguinal mammary fat pads. Metastases models were implanted as single cell suspensions (2–5 × 10^5^ cells per implant) as i.v. or i.p. injections for SKOV3 or BT474 models, or as intra-cardiac (i.c.) injection for MDA-MDA-MB-231-BR-HER2 models, with animals euthanized a maximum of 23 days after implant.

### MRI

MRI experiments were performed at the UBC MRI Research Centre on a 7T Bruker Biospec 70/30 scanner at room temperature with a combination volume (transmit)/surface (receive) coil. DCE-MR imaging data was collected as previously described [19]. Gadovist (Bayer Healthcare) was administered by i.v. catheter as a 5 µL/g bolus dose from 60 mM solution. Macromolecular contrast agent hyperbranched polyglycerol (HPG-GdF, 500 kDa) was synthesized as previously described [20, 21] and administered as a 6 µL/g bolus dose from 100 mg/mL (0.2 mM). Regions of interest (ROI) were drawn on T_2_-weighted RARE images to outline the tumor using ImageJ (NIH) and all other MR analysis was performed using Python. Area Under the Curves (AUC) for Gadovist was determined from the common injection time point to 60 s. A two-parameter linear model was applied to characterize HPG-GdF signal-intensity curves for fractional plasma volume (fPV) determined by the rapid increase at time of injection and for apparent permeability surface area product (aPS) calculated as the slope of later enhancement, as previously described [19]. Both MR and histological modalities imaged slices in the plane perpendicular to an implanted fiducial marker tube to minimize angular differences between MR and histological image slices [22].

### Immunohistochemistry

The general immunohistochemical procedure used has been previously reported [16]. Briefly, 10 µm tumor cryosections were air-dried, imaged for native DiOC_7_(3) or Alexa 546-tagged trastuzumab fluorescence, and fixed in 50% (v/v) acetone/methanol for 10 min at room temperature. Trastuzumab and IgG were visualized in sections using Alexa 546 goat anti-human secondary antibody (Invitrogen). HER2 was subsequently stained with 2.2 × 10^−3^ mg/mL trastuzumab as a primary detection antibody and goat anti-human Alexa 546 secondary. Additional staining was performed using antibodies to PECAM/CD31 (BD PharMingen), pimonidazole (hypoxprobe) collagen IV (Gene Tex) and αSMA (Abcam). Visualization of primary detection antibodies was done using Alexa fluorescence secondary antibodies of appropriate species using 488 nm, 647 nm and 750 nm wavelengths. Nuclear density was stained using Hoechst 33342 (Thermofisher) and imaged at 380 nm.

### Image acquisition and analysis

Sections were imaged as previously described [9] using a system of tiling adjacent microscope fields of view at a resolution of 0.75 µm/pixel. Using ImageJ [23] and user-supplied algorithms, images were superimposed and manually cropped to tumor tissue boundaries with staining artifacts and necrosis removed. False color images were constructed in ImageJ by converting greyscale images to color and overlaying selected layers: trastuzumab (magenta), HER2 (blue or grey), Hoechst 33342 (grey), CD31 (blue), carbocyanine (cyan), pimonidazole (green) and αSMA or CIV (red). Positive fluorescent staining is reported as average intensity (range 1–255) for pimonidazole, trastuzumab and HER2. Perfused vascular density was determined by applying a threshold to CD31 and DiOC7(3) images, with neighboring positive pixels grouped as ‘‘objects’’; CD31 objects with a minimum 20% overlap with DiOC7(3) objects were determined to be perfused vessels. All image pixels were sorted based on their nearest perfused vessel and the average distance is reported as a repeatable measure of vascular density.

### Statistics

All statistical analyses were performed using GraphPad Prism software (version 4.0e for Macintosh). Nonparametric Kruskal Wallis (KW) analysis of variance (ANOVA) for multiple groups and Mann–Whitney U tests were used for comparisons between groups; p values *<0.05, **<0.01 and ***<0.001 are reported. Where appropriate charts display values for individual tumors as means for analysis of whole tumor sections; combined means are reported for 4–8 tumors or tissue discs per group ± standard error (s.e.).

## Results

### Limited access of trastuzumab to micro-metastases of the brain

**Fig. 1:**
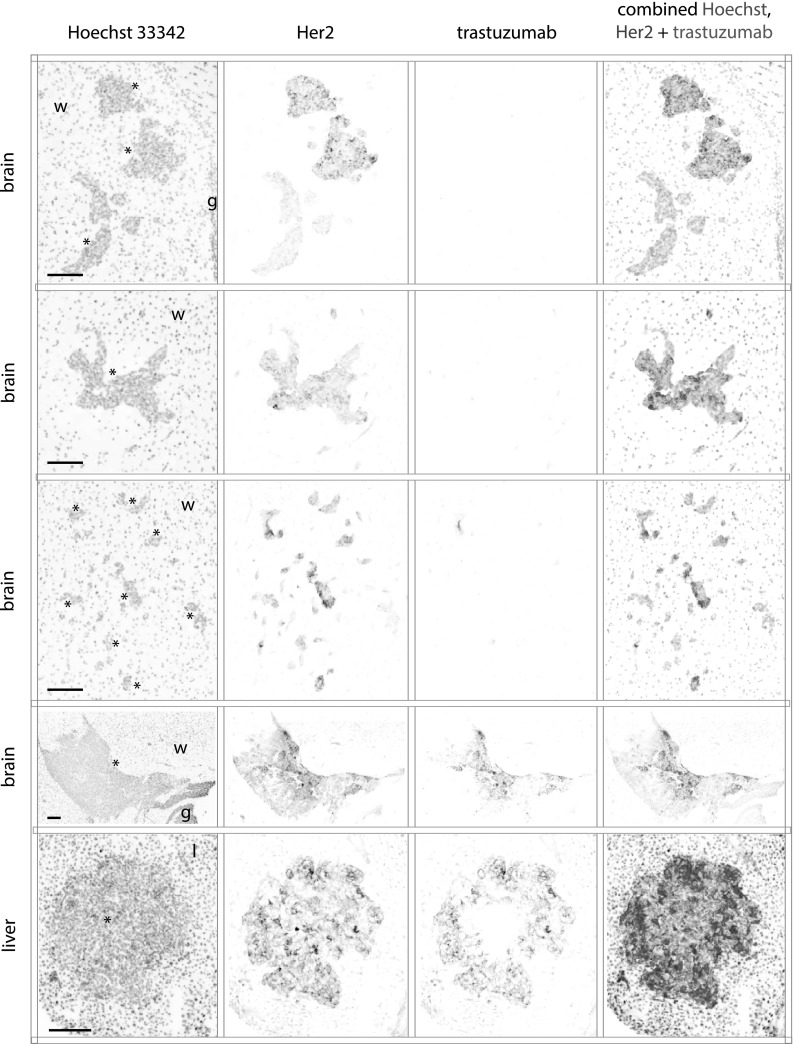
Limited trastuzumab access to metastases of the brain. MDA-MB-231-BR-HER2 cells were implanted by intra-cardiac injection and grew as metastases in the brains and liver of NOD/SCID mice. Mice were treated with 5 mg/kg trastuzumab and tissues collected at 3 h. Fluorescent images of stained cryosections are inverted and shown in grey scale for Hoechst 33342 nuclear dye, HER2 receptor and bound trastuzumab, and all are overlaid in a false color image. Smaller brain micro-metastases (rows 1–3) ranging from small clusters of a few cells to 150 µm in maximum diameter had no detectable bound trastuzumab on the HER2-positive tissues. The largest HER2-positive brain lesion found (row 4) is > 1.5 mm in maximum diameter, and is the only lesion found in the brain with delivered, bound trastuzumab in a heterogeneous pattern. An example metastatic lesion in the liver (row 5) has trastuzumab access from the outside, distributing towards the centre. All scale bars 150 µm. (Color figure online)

MDA-MB-231-BR-HER2 cells inoculated via i.c. implant grow as metastases of varying size in the brain and liver. Animals were treated with a single administration of trastuzumab at 5 mg/kg. Micrometastases with diameters < 150 µm in the brain show no bound trastuzumab despite expression of HER2 (Fig. 1). A larger brain lesion closer to 500 µm in diameter does have bound trastuzumab in a heterogeneous pattern. Liver lesions found in the same animals all have bound trastuzumab present.

**Fig. 2:**
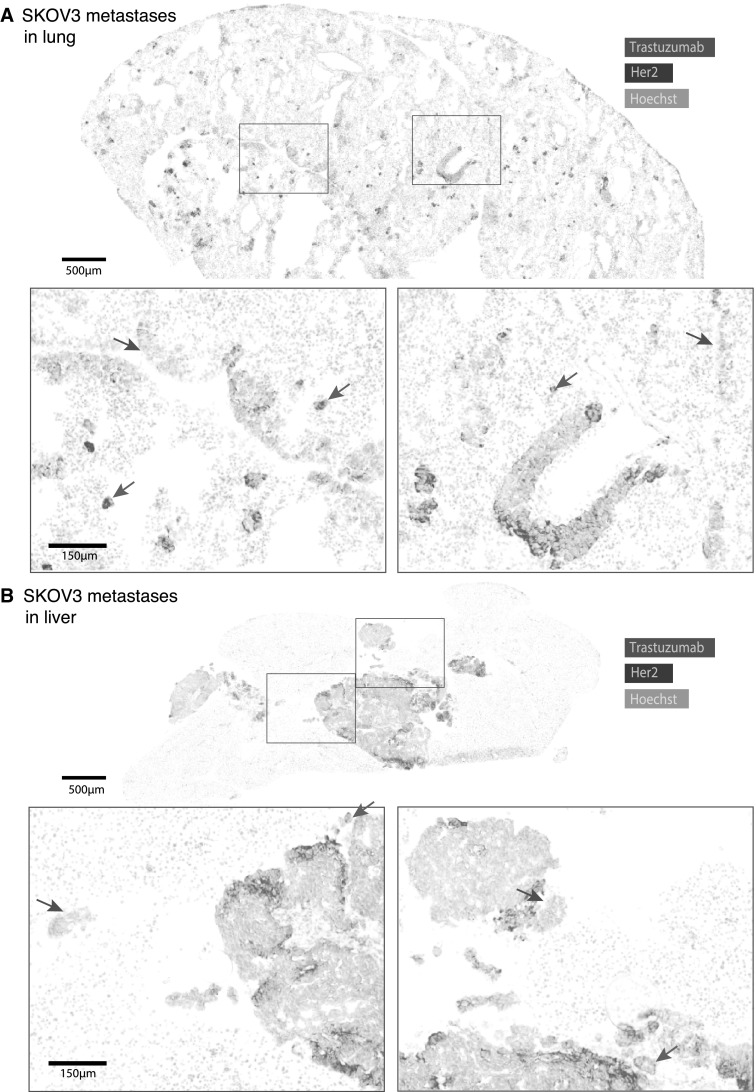
Trastuzumab access to SKOV3 metastases of lung and liver. HER2-positive human SKOV3 ovarian carcinoma cells colonized the lungs of NOD/SCID mice following intravenous (i.v.) injection (**a**) or the liver following intra-peritoneal (i.p.) implantation (**b**), forming metastases of varying sizes. Mice were treated with 10 mg/kg trastuzumab (i.p.) and tissues collected at 20 h. Composite images depict HER2-positive cells (blue), Hoechst 33342 nuclear dye used to stain tissue sections (grey) and the location of bound trastuzumab (magenta). Very small metastases, particularly in the lung, are seen with trastuzumab bound to all extracellular surfaces (green arrows) while others have more heterogeneous trastuzumab distribution with areas of unexposed HER2-positive cells, and others may have very little or no detectable, bound trastuzumab (red arrows). Larger metastases formed in the liver, where microregional heterogeneity is evident despite drug access from both peritoneal and microvascular sources. (Color figure online)

A second metastasis model of SKOV3 cells grown in the lungs (Fig. 2a) or peritoneal cavity on the liver (Fig. 2b) also exhibits a heterogeneous pattern of metastatic growth and trastuzumab binding. Despite being surrounded by regions of highly perfused normal lung and liver tissues there persist regions of tissue that are devoid of bound trastuzumab. Some very tiny lesions in the lung have trastuzumab bound to all extracellular HER2-expressing surfaces (green arrows), while other, larger nodules have very little of the bound antibody (red arrows). Large and small metastases found in the liver all exhibit microregionally heterogeneous distribution patterns of trastuzumab, but most are found to have at least a small amount of bound drug.

### Extravascular distribution of trastuzumab measured in 3D tissue discs is not dependent on degree of HER2 expression

**Fig. 3:**
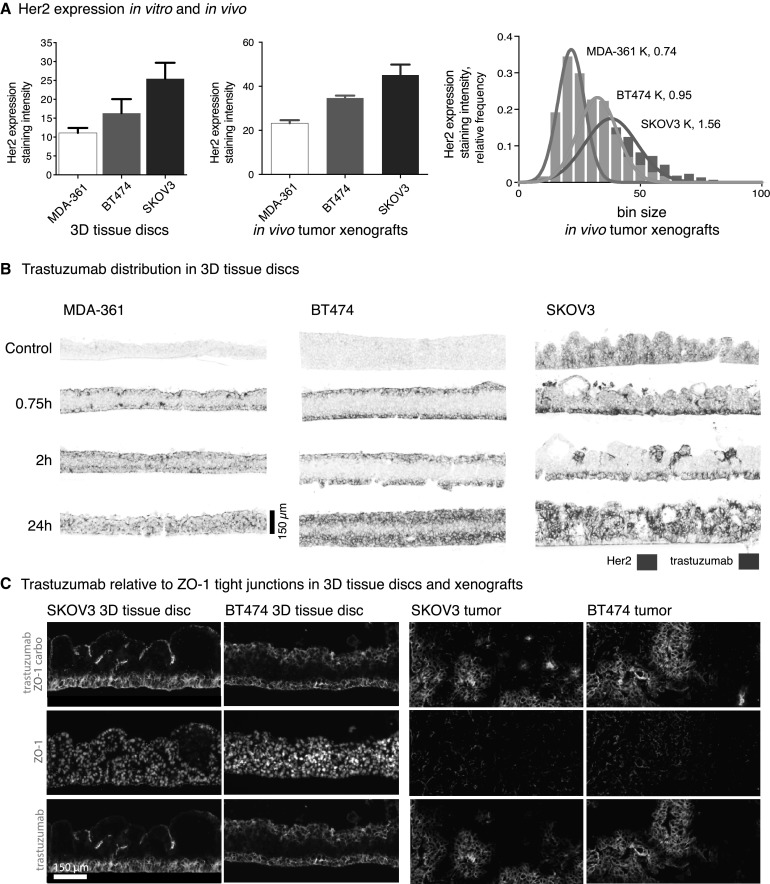
Distribution of MAbs in 3D tissue models in vitro. **a** Relative HER2 expression in human cancer models in vivo as 3D tissue discs (left) and in vivo as xenografted tumors in mice (middle) shows a similar pattern of MDA-MB-361 tissues with the least and SKOV3 tissues with the most HER2 staining. Distribution of HER2 staining also varies within tumors, with SKOV3 tumors having a wider distribution of intensities (right). **b** 3D tissue discs stained for trastuzumab and HER2 are oriented to show their basolateral side down and luminal sides up; inserts were immersed in media containing 50 µg/ml trastuzumab that accessed the tissues from both sides. Immunohistochemical staining is shown as false color images of bound trastuzumab (magenta) relative to HER2 expression (blue). Trastuzumab distributes through the tissue discs at similar rates despite varying HER2 expression in different models, reaching 150 µm within 24 h. SKOV3 tissue discs had limited distribution of trastuzumab from the lumen side. **c** Staining for tight junctions (ZO-1, red) shows a continuous barrier at the luminal side of SKOV3 tissue discs that is not present in BT474 discs, blocking access to trastuzumab. Similar staining of the models in vivo does not show a consistent pattern of higher ZO-1 staining in areas with poor trastuzumab access in either SKOV3 or BT474 tumors. (Color figure online)

The ability of trastuzumab to distribute through tissues in the extravascular compartment was investigated in vivo. HER2-expressing cancer cells were grown as 3D tissue discs on porous PTFE support membranes and as in vivo xenografts and then immunostained for HER2 expression. HER2 staining intensity varies between models but has similar relative variation for in vivo and in vivo comparisons (Fig. 3a); KW ANOVA *p < 0.05 for tissue discs and **p < 0.005 for xenograft models. MDA-MB-361 tissues have the least HER2 overall relative to BT474 and SKOV3, while SKOV3 has the most HER2 staining and the widest distribution of intensities in xenografts. Tissue discs were immersed in stirred media containing 50 µg/mL trastuzumab, with drug access from luminal and basolateral sides of the discs. All models show trastuzumab staining increasing from the disc surfaces towards the centers over time with exception to SKOV3 discs where very little trastuzumab distributes from the luminal side (Fig. 3b). The distance of trastuzumab distribution from the basolateral side is similar in all three models at 0.75 h and 2 h despite HER2 intensity variations. Even with access of trastuzumab being limited to the basolateral side, the SKOV3 discs have trastuzumab bound at distances > 150 µm by 24 h. Staining for tight junction marker ZO-1 shows a continuous barrier on the luminal side of the SKOV3 discs that appears to limit access of trastuzumab on that side; this tight junction barrier is not seen in BT474 discs (Fig. 3c). Staining of ZO-1 in the same tissue models grown as xenografts and treated with 10 mg/kg trastuzumab for 24 h show patent blood vessels that may or may not have trastuzumab bound to perivascular cells. ZO-1 staining is not greater nor more organized around vessels that do not have trastuzumab staining, suggesting these tight junctions are not responsible for the heterogeneous patterns of trastuzumab distribution seen in vivo.

### Measures of vascular density, architecture and function do not consistently correlate with heterogeneous patterns of trastuzumab distribution

**Fig. 4:**
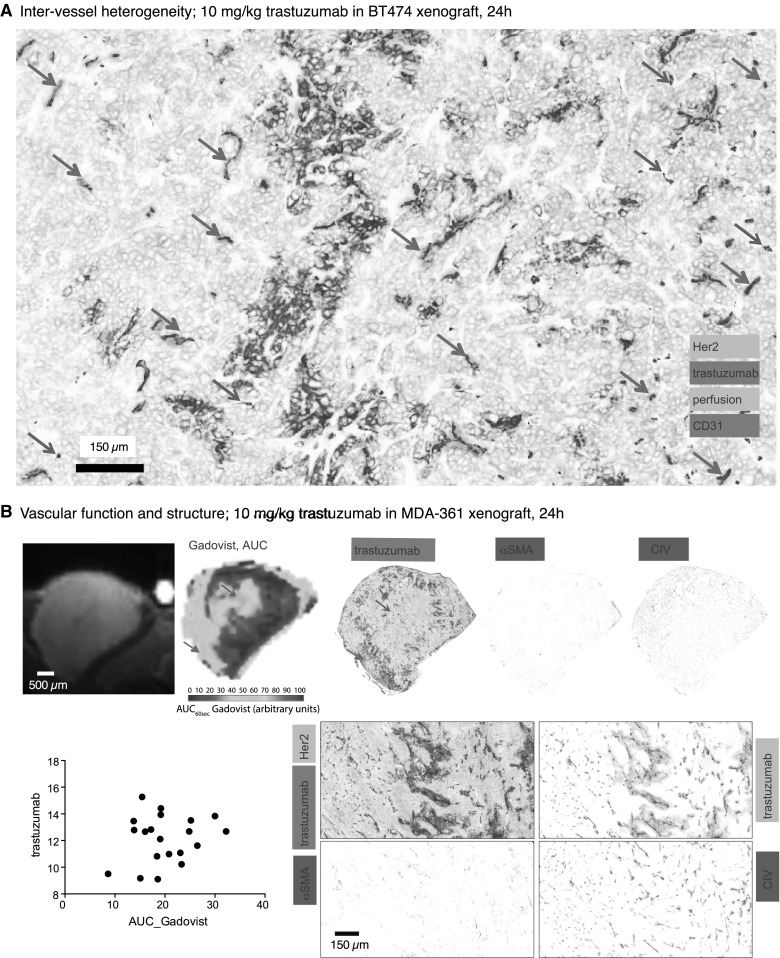
Distribution of Tz in vivo relative to tumor blood vessels. **a** Magnified region of a BT474 xenograft grown orthotopically in inguinal mammary fat pad and treated with 10 mg/kg trastuzumab for 24 h. Trastuzumab extravasates from vessels heterogeneously, with many patent vessels showing no extravascular bound trastuzumab (green arrows) even when adjacent to other patent vessels that do have perivascular trastuzumab. Carbocyanine fluorescent dye (cyan) around CD31 stained vessels (blue) indicates patency; non-patent vessels are indicated (red arrows). **b** DCE-MR imaging of Gadovist accumulation (Gadovist_AUC60_) in MDA-MB-361 tumors is compared with detailed histology mapping of matched histological sections. Regions with greatest AUC_60_(Gadovist), reflecting greatest vascular function, do not consistently correspond to areas of greater trastuzumab distribution (red arrow); this is also demonstrated quantitatively where matched slices were plotted for amount of bound trastuzumab and AUC_60_(Gadovist). The same sections were stained for vascular architectural markers αSMA and CIV (both shown in red), neither of which exhibit a pattern of distribution similar to the presence or absence of trastuzumab (whole sections top; zoomed in region below). (Color figure online)

Significant inter-vessel heterogeneity in trastuzumab distribution is seen in orthotopic BT474 xenografts, with neighboring patent vessels often showing variable amounts of trastuzumab bound to perivascular cells (Fig. 4a), similar to previous findings in MDA-435-LCC6 vector-overexpressing HER2-positive tumors [16]. Non-patent vessels (red arrows) never have extravascular trastuzumab, suggesting intermittent perfusion is not a significant mechanism for reduced trastuzumab access. The same inter-vessel heterogeneity is seen in MDA-MB-361 tumors where vascular function dynamics were further characterized by imaging MR contrast agent Gadovist accumulation (Fig. 4b). Matched histology slices were stained for the location of trastuzumab, vascular patency and markers of vascular maturity CIV and αSMA. Some regions are high for MR-imaged Gadovist uptake, reflecting high vascular function, but these do not consistently co-register with regions of high trastuzumab distribution in histology sections. A quantitative correlation between Gadovist AUC and trastuzumab in matched image slices obtained from multiple tumors was not evident. Similarly, neither αSMA nor CIV are present to a greater degree proximal to vessels with or without trastuzumab bound to perivascular cells.

### Trastuzumab distribution increases with time and dose in vivo and shows considerable inter-tumor and intra-tumor heterogeneity

**Fig. 5:**
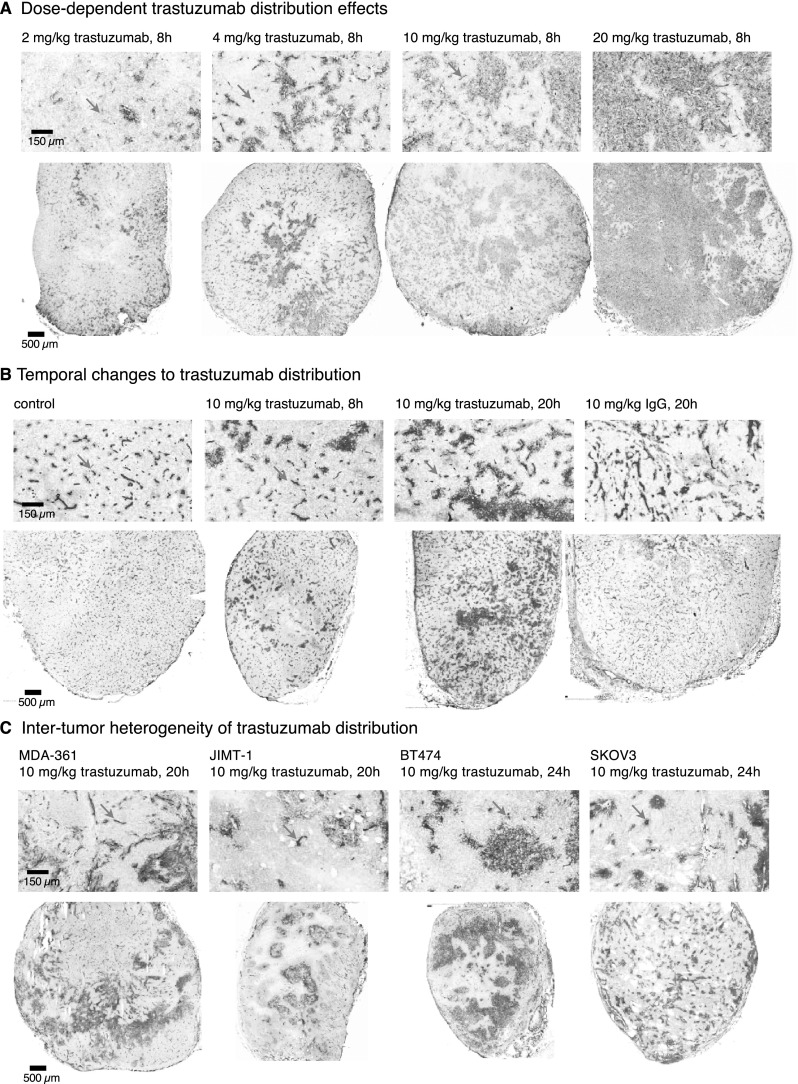
Inter- and intra-tumor heterogeneity of trastuzumab distribution in HER2-positive xenografts. **a** Greater doses of trastuzumab result in increased accumulation in BT474 xenografts, with trastuzumab found around more patent vessels. However some heterogeneity persists even at 20 mg/kg, a dose that is 5× the typical clinical loading dose of trastuzumab. **b** Trastuzumab accumulation increases with increased exposure time as shown in the BT474 orthotopic xenografts, where the MAb is extravasating from more vessels at longer timepoints. Similar exposure to humanized isotype control antibody IgG suggests the non-targeted IgG has a more homogeneous distribution through the tissues (right). Labeling of hypoxia via pimonidazole staining (green) illustrates majority of hypoxic regions do not have overlapping trastuzumab stain (magenta), although this is not exclusive. Regions without hypoxia also show limited trastuzumab distribution. **c** Naturally HER2-overexpressing xenograft models of breast (BT474, JIMT-1, MDA-361) and ovarian (SKOV3) tumors grown in NOD/SCID mice were treated with 10 mg/kg trastuzumab by ip injection with tumors collected at 20–24 h. Significant heterogeneity is seen, with substantial amounts of HER2-positive tissue remaining unbound for trastuzumab in all four models. HER2-positive staining (grey) shows the relative homogeneity of receptor expression; large negative staining areas are necrosis or tissue gaps. Perfused vessels (carbocanine; cyan) that have an absence of perivascular bound trastuzumab are found in each model, at every dose level and at all timepoints (orange arrows). (Color figure online)

The proportion of tumor vessels with perivascular trastuzumab can be increased by dose and time. Trastuzumab accumulation is dose responsive with greater distribution in BT474 tumors at each increase in dose from 2 to 20 mg/kg at 8 h (Fig. 5a). Trastuzumab accumulation also increases with time, where at 24 h it is extravasating from more vessels than at 8 h after a 10 mg/kg dose; comparatively, the isotype control IgG antibody is seen proximal to nearly all vessels in the same BT474 tumor model at 20 h (Fig. 5b). However, areas of HER2-positive tumor tissue are consistently found that persist with poor trastuzumab access at highest doses and peak exposure times. Regions of BT474 tumor tissue with particularly poor blood flow are shown via pimonidazole labeling of hypoxia (Fig. 5b). Trastuzumab and hypoxia rarely overlap in these tumors, however trastuzumab is not always excluded from hypoxic regions and non-hypoxic regions are not always positive for trastuzumab. Examples of hypoxia relative to trastuzumab binding in additional tumor models are shown in Supplementary Fig. 1.

Inter-tumor and inter-model heterogeneity of trastuzumab distribution is also illustrated in Fig. 5c with examples of MDA-MB-361, JIMT-1 and BT474 breast cancers and SKOV3 ovarian carcinoma, all showing significant areas of HER2-positive tissue without bound trastuzumab. The microregional patterns of trastuzumab distribution vary considerably; some tumors exhibit large central areas of tissue that are devoid of trastuzumab, others have their greatest distribution originating in the center of the tumors. Others have a more homogeneous distribution throughout the tumor, though many vessels persist without any extravasating drug. Selected images represent the diverse range of trastuzumab distribution patterns and are not necessarily representative of particular models. Considerable inter-vessel heterogeneity with respect to the amount of bound trastuzumab in perivascular tissues is observed in all the models, as is emphasized in magnified regions.

Even after repeated, high doses of trastuzumab, small but potentially significant regions of tumors can be found with little trastuzumab access (Supplemental Fig. 2). Tumor image maps illustrate that the microregional distribution of trastuzumab is highly heterogeneous at the vessel level, with no consistent or predictable patterns currently discernible.

### Dynamic vascular permeability and blood volume measurements do not consistently relate to patterns of trastuzumab distribution

**Fig. 6:**
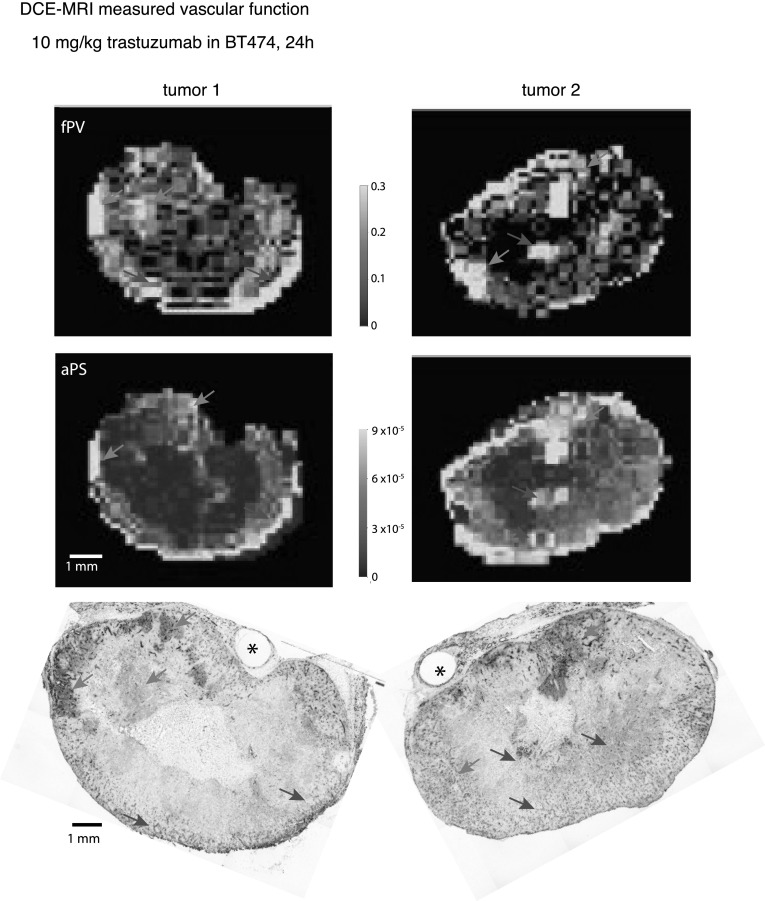
Dynamic measurements of vascular function relative to trastuzumab distribution. BT474 tumors were imaged in 7T Bruker MRI and uptake of HPG-GdF contrast agent (500 kDa) measured. Parameter maps show calculated values for fractional plasma volume (fPV) and apparent permeability surface area (aPS), reflecting vascular perfusion and permeability respectively. Matched histology sections are stained for bound trastuzumab (magenta) administered at 10 mg/kg 24 h prior to imaging and tissue collection, and for HER2 (grey), carbocyanine marker of perfusion (cyan) and for CD31 vasculature (blue). Areas of vascular function (MRI) and trastuzumab (histology) correlation are indicated (orange arrows) in both modalities; example areas of poor matching are also shown (red arrows). Stars indicate location of fiducial markers for multi-modal slice comparison. (Color figure online)

The histological measure of perfusion using carbocyanine is a useful indication of vessel patency, however it is static and therefore its interpretation is limited. The role of vascular function on trastuzumab distribution was further investigated using dynamic contrast enhanced MRI (DCE-MRI) of a high molecular weight contrast agent, HPG-GdF (MW 500 kDa) (Fig. 6). As previously described, repeat imaging of contrast agent presence in the tumors is analysed and the initial appearance of HPG-GdF reflects fPV and its accumulation over time indicates the apparent permeability surface area (aPS) [19]. Tumors were excised immediately after imaging; corresponding histological sections are compared to the DCE-MRI derived parameter maps. BT474 tumors have microregionally variable levels of both fPV and aPS, each exhibiting regions of distinction. Regions of high fPV are well matched by histological images of carbocyanine that indicate areas of very high perfusion but some of these regions do not have any significant accumulation of trastuzumab. The reverse can also be found, where regions with relatively low fPV correspond to high trastuzumab. Similarly, there are some significant trastuzumab accumulation areas that have relatively low aPS while some high aPS values correspond to areas with relatively low trastuzumab. Examples of good or bad correlation between vascular function and trastuzumab distribution are highlighted with arrows and suggest that neither of the MRI-derived parameters consistently or adequately explain microregional distribution of trastuzumab.

### Dramatic decrease in trastuzumab distribution when combined with VEGF ablation

**Fig. 7:**
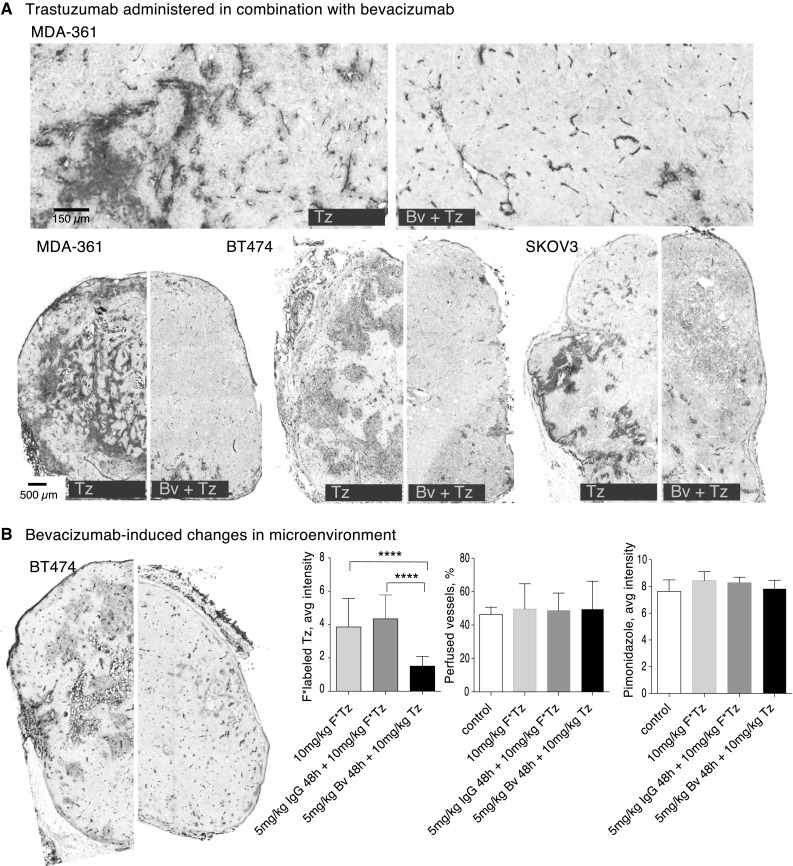
Bevacizumab decreases subsequent distribution of trastuzumab. Animals bearing MDA-MB-361, BT474 or SKOV3 tumors were treated with 5 mg/kg bevacizumab (Bv) for 48 h prior to 10 mg/kg doses of fluorescent-labeled trastuzumab (Tz). **a** The amount of trastuzumab (magenta) accumulation in the xenografts is reduced despite persistence of HER2 expression (grey) when Tz administration follows pre-treatment with Bv. The number and distribution of perfused vessels (carbocyanine, cyan; CD31, blue) remains similar, but fewer of these patent vessels have bound perivascular trastuzumab. **b** Reduced trastuzumab accumulation occurs despite absence of change in the density of perfused vessels or in the presence of poorly oxygenated tissues measured by staining for pimonidazole (green). (Color figure online)

Despite poor correlation with histological and MRI-derived parameters of vascular architecture and function with trastuzumab distribution, the significant inter-vessel heterogeneity seen in tumors with incomplete trastuzumab distribution suggests a role for vascular permeability or extravasation of the MAb from vessels. This was further explored using anti-angiogenic agent bevacizumab, which binds VEGF-A and decreases vascular permeability. A single 5 mg/kg dose of bevacizumab given 48 h prior to subsequent trastuzumab treatment causes a significant reduction in the number of vessels with perivascular trastuzumab, thereby also a decrease in the overall amount of bound trastuzumab found in tumors, relative to those treated with an isotype control antibody (Fig. 7). This effect is seen in multiple HER2-positive models, and occurs despite the absence of measureable differences in vascular perfusion or hypoxia occurring in these tumors. A bevacizumab-mediated decrease in subsequent trastuzumab accumulation occurs to a similar degree at higher (10 mg/kg) and lower (2.5 mg/kg) single doses of bevacizumab, and in tumors treated with repeat dosing up to 2 weeks in duration (Supplemental Fig. 3).

## Discussion

This study has shown direct evidence of poor trastuzumab access to metastases of the brain in a preclinical model. Small clusters of cells that are immediately surrounded by highly perfused brain, liver or lung tissues may remain incompletely or in some cases totally unbound for trastuzumab. Some avascular metastases of the brain may be outside the BBB and therefore have poor access to trastuzumab, while larger lesions with active angiogenesis and blood vessel support may have a blood-tumor barrier (BTB) with greater trastuzumab delivery, however we have shown that microregional distribution of trastuzumab can remain highly heterogeneous and incomplete even when delivered by vessels within HER2-positive lesions. Our data are in agreement with other studies evaluating the permeability of brain metastases, which have found no correlation between metastases size, vessel density, aggressiveness or morphology and their permeability [5, 24]. Mittapalli et al. have used mathematical modeling and MRI data to show a limit in the vascular pore size of MDA-MB-231-BR-HER2 metastases that would preclude access to MAbs including trastuzumab [25]. Other groups have also shown poor trastuzumab access in this model [26, 27]. As found here, trastuzumab has been detected in larger brain lesions in murine models [28] where higher systemic doses of the MAb were required to achieve anti-cancer efficacy seen for tumors grown in the mammary fat pad. Accumulation over time has also been noted in patient metastases including those of the brain, where labeled antibodies were used for diagnostic purposes using PET [29, 30]. It is unknown if human tumors or metastases exhibit the same barriers to MAb access. As we have shown, access of trastuzumab to metastases and the distribution within can be highly heterogeneous, and is likely to be highly model and patient specific. Ultimately, a majority of patients treated with trastuzumab eventually experience progression and relapse. The rate of brain metastases has increased for HER2-positive patients following treatment with trastuzumab, suggesting the MAb therapeutic is less effective for tumors ‘behind’ the BBB than in metastases elsewhere such as the liver and lung [6]. Here we show direct evidence of poor trastuzumab access to HER2-positive brain metastases and suggest that this confirms non-uniform access of MAbs to cancers and metastases may be driving a significant portion of resistance to their effects.

Using tumor mapping analysis and DCE-MRI we further examined the impacts of HER2 expression, tumor blood vessel architecture, vascular function and the tumor microenvironment on the patterns of distribution of trastuzumab in primary and metastatic models. We have observed that even when trastuzumab does have access to tumor tissues, the pattern of distribution is highly heterogeneous, similar to our previous work in a vector-overexpressing HER2-positive breast cancer model [16]. Other groups demonstrating a limitation on the accumulation of trastuzumab in preclinical tumors have focused on net tumor accumulation and suggest that a major limitation to MAb distribution is their difficulty distributing through the solid tumor tissue in the extravascular space [13, 14, 17]. In humans MAbs have a long half-life and are administered for many months. The pharmacokinetic parameters of MAbs are therefore thought to likely accommodate relatively slow distribution of MAbs through the interstitium [14, 31]. However, our 3D tissue disc data shows trastuzumab is able to diffuse relatively well through tumor tissues in vivo in conditions of poor convective flow similar to an environment with high interstitial fluid pressure in vivo. Neither is trastuzumab distribution limited by tight binding to antigen proximal to vessels as suggested by the binding-site barrier hypothesis [15], as reaches distances > 150 µm from sources within 24 h despite high HER2 expression. This distance is similar to the diffusion limit for oxygen, therefore most tumor tissues are within this 150 µm range of a blood vessel and could be expected to have adequate exposure. These data suggest that neither the BSBH nor high interstitial fluid pressure adequately explain microregional variation in trastuzumab binding. Given the incomplete access of trastuzumab seen in our models, other vessel- and tissue-level barriers appear to limit its distribution in the tumor microenvironment.

One barrier to MAb distribution appears to be the presence of tight junctions, labeled using ZO-1. However, these continuous barriers are not seen in vivo where inter-vessel heterogeneity occurs and even neighboring perfused vessels exhibit dramatic differences in the amount of bound drug on their perivascular cells. Vascular maturity and architecture has also been attributed to poor drug access [32], however, no correlation between markers for aSMA or CIV and the relative distribution of trastuzumab has been found.Higher doses and longer exposures do lead to higher numbers of vessels with perivascular trastuzumab, suggesting that some degree of intermittent perfusion or very poorly permeable vessels impact the observed microregional heterogeneity. Non-perfused vessels have not been observed to have trastuzumab on perivascular cells, which suggests intermittent perfusion is an unlikely mechanism for observed inter-vessel heterogeneity. Dynamic measurements of perfusion and permeability derived from DCE-MR imaging of high molecular weight contrast agent HPG-GdF (500 kDa) suggest the intuitive relationship between vascular function and drug access is important, but does not consistently explain the heterogeneous patterns of drug distribution seen. Areas of highly perfused tissue marked by carbocyanine in histological sections correspond to areas of high perfusion and/or permeability in MRI. However, these tumor areas of high vascular function described in both imaging modalities may or may not have bound trastuzumab. Similarly, regions with substantial amounts of bound trastuzumab do not necessarily correspond to areas of high perfusion or permeability measured using aPS and fPV. HPG-GdF-derived parameters fPV and aPS arise under conditions that are closer to a permeability-limited regime than standard low-molecular weight contrast agents. However, in the highly chaotic vascular environment of tumours we can still expect these MR-derived parameters of vascular function to not be completely independent of each other.

All in vivo models investigated were found to be sensitive to the effects of bevacizumab, a monoclonal antibody that binds VEGF-A and decreases vascular permeability [33]. In our xenograft studies we see a dramatic reduction in the accumulation of trastuzumab in tumors when following pre-treatment with bevacizumab. The majority of tumor blood vessels cease to have extravasated trastuzumab bound to perivascular cells. These detailed images of the microenvironment highlighting reduced distribution at the vessel level agree with previous studies showing average reductions in trastuzumab delivery to the whole tumor [34, 35]. In some cases VEGF ablation changes tumor vasculature, either the density or the fraction of perfused or mature vessels and the resulting overall function of tumor vasculature, but did not do so measurably in these tumors where neither the proportion of perfused vessels nor the amount of tumor hypoxia changed.

The clinical impact of combination treatments is unclear, largely due to the difficulty in measuring drug distribution directly. Addition of bevacizumab did not improve outcomes for metastatic breast cancer patients being treated with docetaxel and trastuzumab [36]. Overall, poor clinical results have been found when combining VEGF ablation therapies with trastuzumab (reviewed in [37]). The potential risks of combining MAbs and anti-angiogenic treatments have been recognized, and many publications and reviews in the pre-clinical and clinical fields of have called for investigations regarding optimal treatment and scheduling regimens for these combinations [37–43].

New strategies that are able to more effectively target and kill cancer cells, particularly metastatic disease, are urgently required. Efforts to vary antigen-binding affinities and the size of MAb fragments have been explored to improve their distribution through the *extravascular* compartment [13, 14]. Our data highlight the importance of also considering access of antibodies to target tumor tissues and whether microregional distribution of MAb therapeutics may be affected when combined with vascular damaging agents or when targeting occult metastases. Improvements to MAb anti-cancer activity could be made in selection of combination therapies and the design of treatment schedules, as well as in the design of novel targeted drugs. Efforts to examine these phenomena in the clinic would be of significant interest.

## Conclusion

We have shown evidence for poor distribution and access of the MAb trastuzumab in preclinical tumors through direct visualization of bound drug, with particular implications for metastatic tumors, including those of the brain. Our data suggest that the tumor microenvironment and tissue- and vessel-level barriers to drug distribution could effectively limit access of the drug to all its target cells. These effects appear to be more important than slow interstitial distribution resulting from high interstitial fluid pressures or high binding affinity to HER2. Further, administering trastuzumab in combination with vascular disrupting agents could significantly impact its activity via reducing access.

## Electronic supplementary material

Below is the link to the electronic supplementary material.

Supplemental Fig. 1**—**Accumulation of trastuzumab relative to regions of tumor hypoxia. MDA-435-LCC6(HER2) (top) and SKOV3 (bottom) xenografts were exposed to 10 mg/kg trastuzumab for 20 and 8 h, respectively. Pimonidazole was administered at 60 mg/kg 2 h prior to tissue collection; composite false color images of staining show bound trastuzumab (magenta), vasculature (CD31, blue; carbocyanine, cyan), pimonidazole (green), HER2 (grey, left and right) and Hoechst 33342 (grey, centre). Many hypoxic, pimonidazole-positive areas are negative for trastuzumab however some areas of overlapping pimonidazole and trastuzumab are found in each model. Not all trastuzumab-negative areas are hypoxic, suggesting poor access of trastuzumab is not limited to regions with poor vascular function (PDF 14053 KB)

Supplemental Fig. 2—Accumulation of trastuzumab following prolonged exposure. BT474 (top) and MDA-MB-361 (bottom) xenografts were exposed to trastuzumab for 10 days with the relatively high 10 mg/kg dose of trastuzumab (2.5× clinical loading dose) administered every 3 days, with the last dose 24 h before tissue collection. Tumor maps show staining for bound trastuzumab (magenta), vasculature (CD31, blue; carbocyanine, cyan) and HER2 (grey). The majority of BT474 tumors are positive for bound trasutuzmab, however perfused vessels with no perivascular trastuzumab staining can still be found (orange arrows). MDA-MB-361 tumors exhibit less trastuzumab binding than is seen in single dose tumors, possibly due to downregulation of the HER2 receptor following the prolonged exposure. Small areas of HER2-positive tissue that does not have bound trastuzumab are also found in these tumors, and are most often found to be avascular nodules with poor distribution of trastuzumab coming from the periphery (PDF 1971 KB)

Supplemental Fig. 3—Reduced trastuzumab distribution when combined with multiple doses of bevacizumab. (A) The amount of trastuzumab accumulation in HER2-positive xenografts (magenta; unbound HER2 in grey; carbocyanine in cyan; CD31 in blue) is reduced when administration follows pre-treatment with bevacizumab administered as single 2.5, 5 or 10 mg/kg doses for 48 h prior to trastuzumab treatment. Repeat dosing of 2.5 mg/kg bevacizumab for 1-2 weeks also causes reduced trastuzumab distribution. (B) The degree of trastuzumab decrease is similar at all doses despite the absence of other changes in the tumor microenvironment such as the density of perfused CD31 vessels (average distance of tumor tissue to nearest carbocyanine-perfused vessel) or the existence of poorly vascularized hypoxic tissue measured as the amount of pimonidazole labeling (PDF 952 KB)

## Abbreviations

aPS: Apparent permeability surface area
AUC: Area under the curve
BBB: Blood–brain barrier
BSBH: Binding site barrier hypothesis
Bv: Bevacizumab (Avastin)
DCE-MRI: Dynamic contrat enhanced magnetic resonance imaging
fPV: Fractional plasma volume
HPG-GdF: Hyperbranched polyglycerol macromolecular contrast agent
MAb: Monoclonal antibody
PTFE: Polytetrafluoroethylene
ROI: Region of interest
Tz: Trastuzumab (Herceptin)
