# Supplementary figures and images for: Heterogeneous distribution of trastuzumab in HER2-positive xenografts and metastases: role of the tumor microenvironment
Source: Clin Exp Metastasis. 2018 Sep 8;35(7):691–705. doi: 10.1007/s10585-018-9929-3 (PMC6209006; doi:10.1007/s10585-018-9929-3)

Trastuzumab relative to hypoxia / vascular function

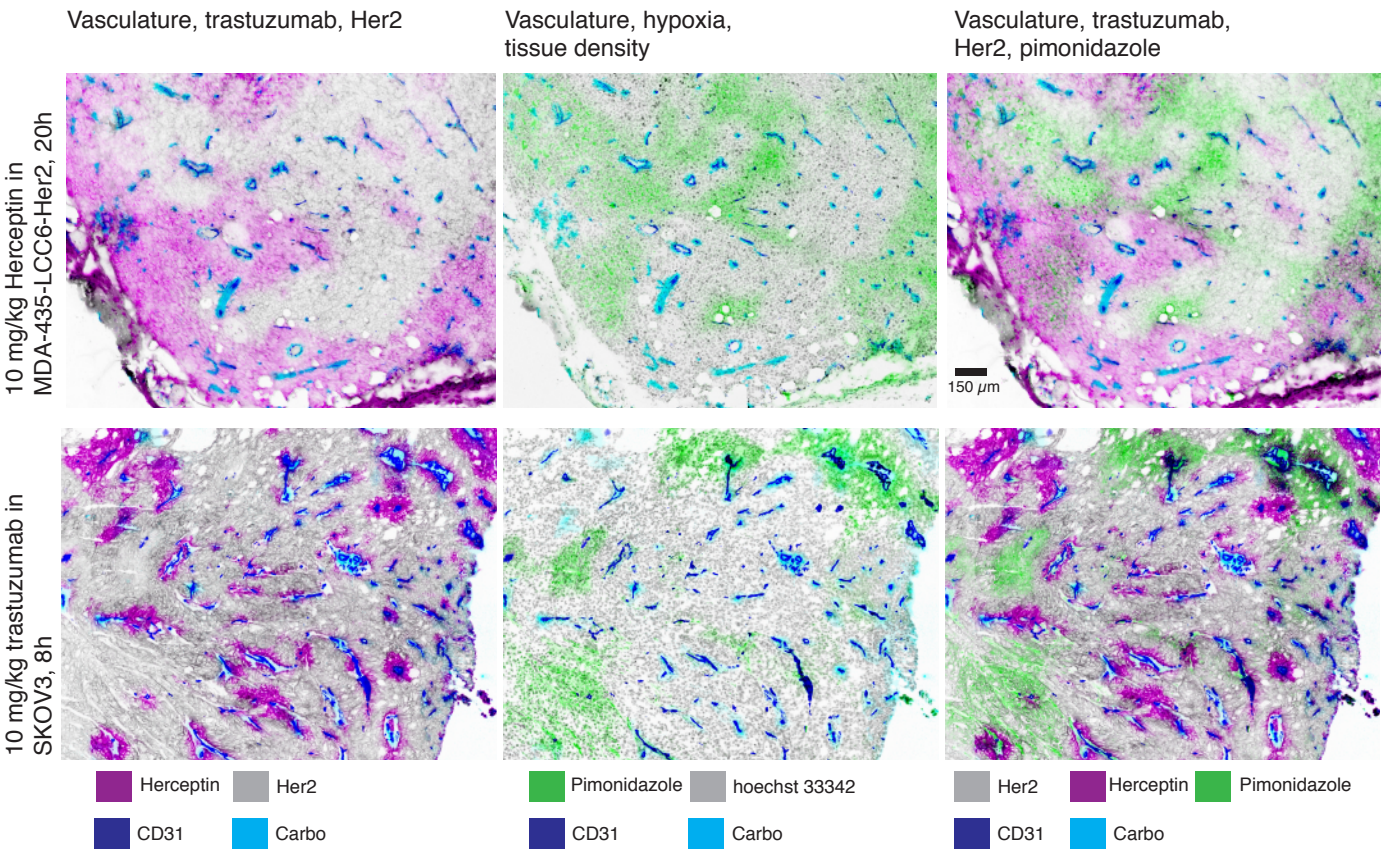

Supplementary Figure 1

Supplement: Supplementary file 1 — Supplemental Fig. 1—Accumulation of trastuzumab relative to regions of tumor hypoxia. MDA-435-LCC6(HER2) (top) and SKOV3 (bottom) xenografts were exposed to 10 mg/kg trastuzumab for 20 and 8 h, respectively. Pimonidazole was administered at 60 mg/kg 2 h prior to tissue collection; composite false color images of staining show bound trastuzumab (magenta), vasculature (CD31, blue; carbocyanine, cyan), pimonidazole (green), HER2 (grey, left and right) and Hoechst 33342 (grey, centre). Many hypoxic, pimonidazole-positive areas are negative for trastuzumab however some areas of overlapping pimonidazole and trastuzumab are found in each model. Not all trastuzumab-negative areas are hypoxic, suggesting poor access of trastuzumab is not limited to regions with poor vascular function (PDF 14053 KB) [file 10585_2018_9929_MOESM1_ESM.pdf]

BT474;  
(10 mg/kg trastuzumab 72h x3) + 10 mg/kg 24h

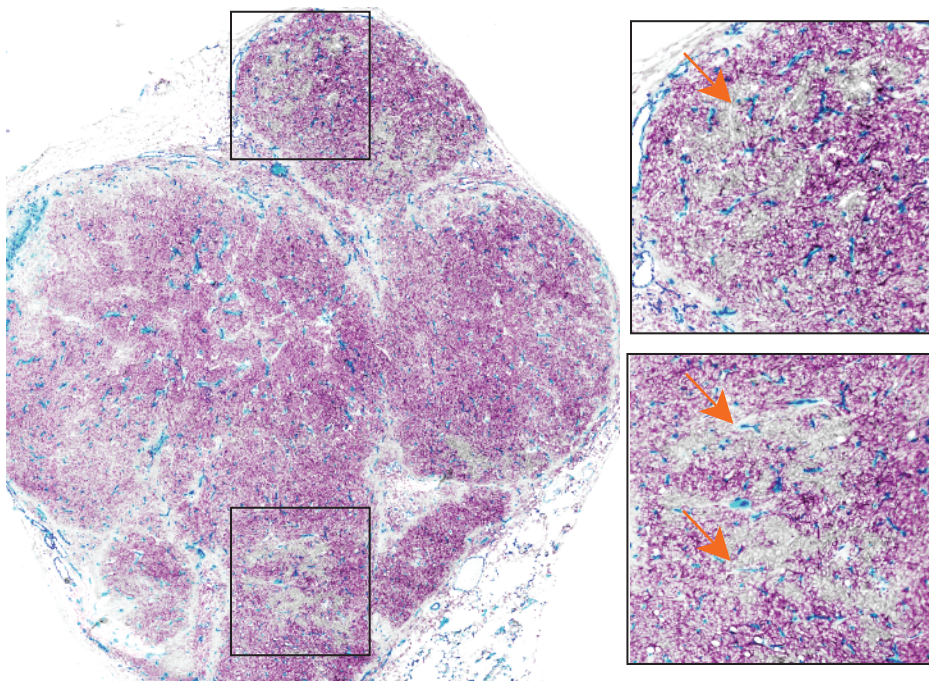

MDA-361;  
(10 mg/kg trastuzumab 72h x2) + 10 mg/kg 24h

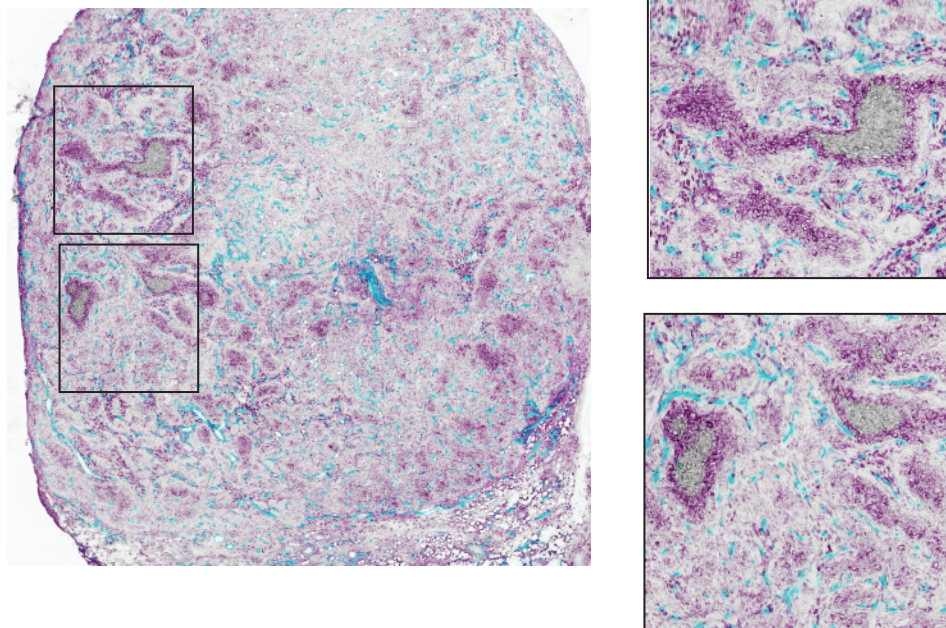

Supplementary Figure 2

Supplement: Supplementary file 2 — Supplemental Fig. 2—Accumulation of trastuzumab following prolonged exposure. BT474 (top) and MDA-MB-361 (bottom) xenografts were exposed to trastuzumab for 10 days with the relatively high 10 mg/kg dose of trastuzumab (2.5× clinical loading dose) administered every 3 days, with the last dose 24 h before tissue collection. Tumor maps show staining for bound trastuzumab (magenta), vasculature (CD31, blue; carbocyanine, cyan) and HER2 (grey). The majority of BT474 tumors are positive for bound trasutuzmab, however perfused vessels with no perivascular trastuzumab staining can still be found (orange arrows). MDA-MB-361 tumors exhibit less trastuzumab binding than is seen in single dose tumors, possibly due to downregulation of the HER2 receptor following the prolonged exposure. Small areas of HER2-positive tissue that does not have bound trastuzumab are also found in these tumors, and are most often found to be avascular nodules with poor distribution of trastuzumab coming from the periphery (PDF 1971 KB) [file 10585_2018_9929_MOESM2_ESM.pdf]
