# Supplementary material for: Heterogeneous distribution of trastuzumab in HER2-positive xenografts and metastases: role of the tumor microenvironment
Source: Clin Exp Metastasis. 2018 Sep 8;35(7):691–705. doi: 10.1007/s10585-018-9929-3 (PMC6209006; doi:10.1007/s10585-018-9929-3)
Supplement: Supplementary file 3 — Supplemental Fig. 3—Reduced trastuzumab distribution when combined with multiple doses of bevacizumab. (A) The amount of trastuzumab accumulation in HER2-positive xenografts (magenta; unbound HER2 in grey; carbocyanine in cyan; CD31 in blue) is reduced when administration follows pre-treatment with bevacizumab administered as single 2.5, 5 or 10 mg/kg doses for 48 h prior to trastuzumab treatment. Repeat dosing of 2.5 mg/kg bevacizumab for 1-2 weeks also causes reduced trastuzumab distribution. (B) The degree of trastuzumab decrease is similar at all doses despite the absence of other changes in the tumor microenvironment such as the density of perfused CD31 vessels (average distance of tumor tissue to nearest carbocyanine-perfused vessel) or the existence of poorly vascularized hypoxic tissue measured as the amount of pimonidazole labeling (PDF 952 KB) [file 10585_2018_9929_MOESM3_ESM.pdf]

A Trastuzumab administered in combination with bevacizumab: BT474 xenografts

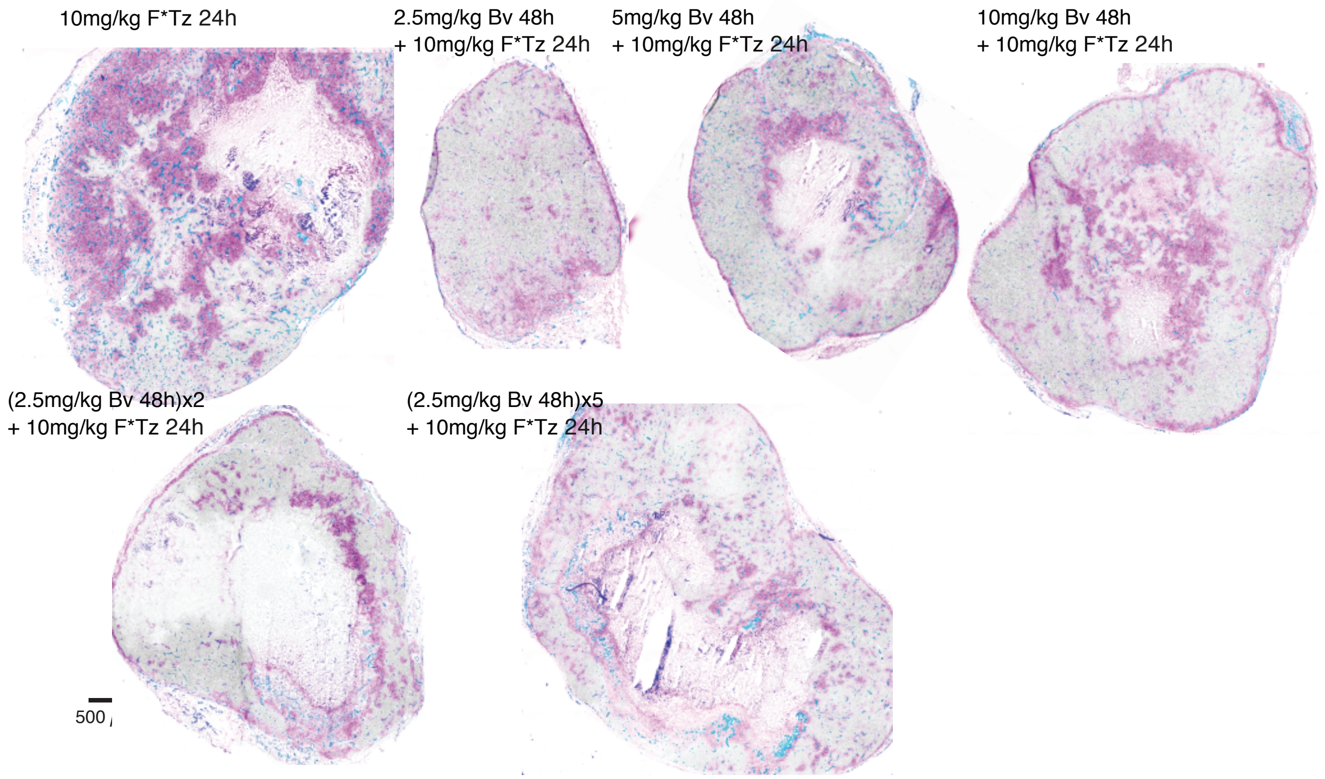

B Bevacizumab-induced changes in microenvironment

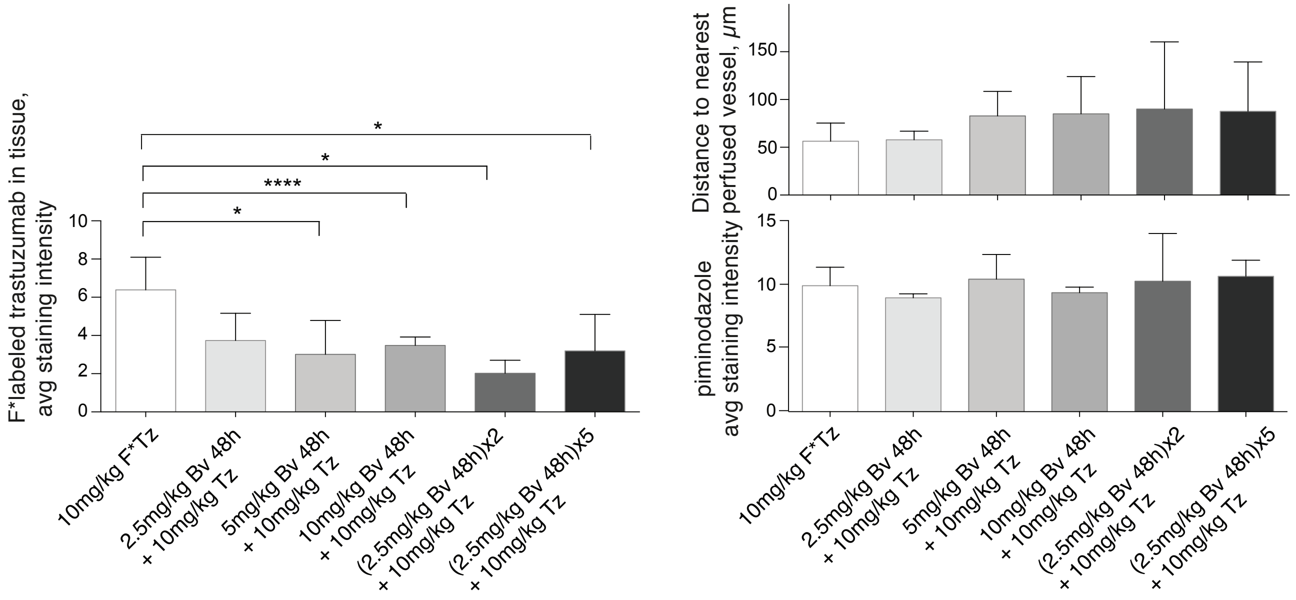

Supplementary Figure 3
